# Machine Learning for Haptics: Inferring Multi-Contact Stimulation From Sparse Sensor Configuration

**DOI:** 10.3389/fnbot.2019.00051

**Published:** 2019-07-10

**Authors:** Huanbo Sun, Georg Martius

**Affiliations:** Autonomous Learning Group, Max Planck Institute for Intelligent Systems, Tübingen, Germany

**Keywords:** haptics, machine learning, multi-contact, sparse sensor network, transfer learning, insufficient data, robotic application

## Abstract

Robust haptic sensation systems are essential for obtaining dexterous robots. Currently, we have solutions for small surface areas, such as fingers, but affordable and robust techniques for covering large areas of an arbitrary 3D surface are still missing. Here, we introduce a general machine learning framework to infer multi-contact haptic forces on a 3D robot's limb surface from internal deformation measured by only a few physical sensors. The general idea of this framework is to predict first the whole surface deformation pattern from the sparsely placed sensors and then to infer number, locations, and force magnitudes of unknown contact points. We show how this can be done even if training data can only be obtained for single-contact points using transfer learning at the example of a modified limb of the Poppy robot. With only 10 strain-gauge sensors we obtain a high accuracy also for multiple-contact points. The method can be applied to arbitrarily shaped surfaces and physical sensor types, as long as training data can be obtained.

## 1. Introduction

Robots can become helpful in more and more application areas if they can robustly interact with the real world and if they are safe for humans. Haptic sensation is a crucial element on the path of developing such robots. Up to now, haptic research advances are mainly on creating touch-sensitive robotic hands (Odhner et al., 2014; Kaboli et al., 2016; Boutry et al., 2018; OpenAI et al., 2018; Ward-Cherrier et al., 2018), surgical robotic systems (Munawar and Fischer, 2016; Peters et al., 2018), and commercial touch screens, such as the in-display fingerprint readers and so forth. These systems have in common that only a small area is equipped with highly precise haptic sensation capabilities. However, there is only little research on large-surface haptics for robotic applications with some recent advances using a grid of small patches (Rogelio Guadarrama-Olvera et al., 2018), flexible haptic skins (Lee, 2017), and machine learning approaches (Sun and Martius, 2018). Haptic feedback at large parts of the body is essential for robots to learn interaction patterns, exploit the environment, detect unexpected or safety-relevant situations for mastering real-world challenges.

Ideally, a haptic system should provide contact parameters, such as contact location and directional force information (e.g., normal and shear forces) for multiple-contact points with high spatial and temporal resolution. In addition, a haptic system should be: robust to long-lasting impacts, low-cost, energy-saving, and computationally inexpensive. In this work, we present a favorable trade-off of the above criteria with a particular emphasis on the needs of large-surface haptic systems. For instance, the location, strength, and number of contact points are more important for large surface haptic applications than texture and slip information which is desired in dexterous manipulation tasks. The application we are aiming at is a robot in a developmental learning setting that needs to learn behavior from interactions. Thus our design aims at whole surface haptic sensation with high robustness and simple hardware.

Existing large-area haptic devices are often bulky, requiring complex manufacturing processes, have high energy consumption and are not robust enough for long-term interaction. Typical approaches use a dense arrangement of physical sensor units (piezoelectric, resistive, capacitive, pressure, infrared, etc.). There are several ways to replace such dense configurations by sparse sensor arrangement reducing hardware complexity and potentially energy consumption while maintaining most of the functionality. This includes optical methods (Koppelhuber and Bimber, 2013) and electrical impedance tomography methods (Lee, 2017).

**Figure 1:**
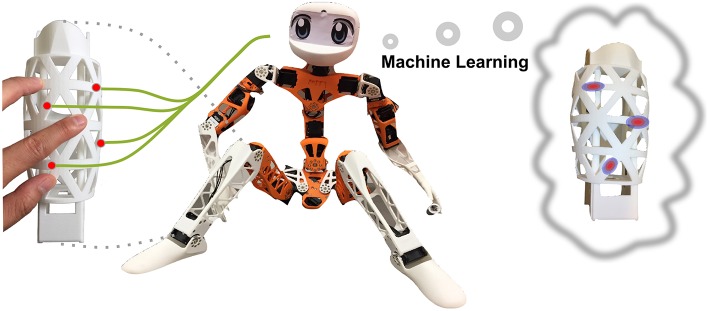
Overall goal of the method: inferring multi-contact haptic stimulation from a few sensors measuring internal deformation.

This paper proposes a haptic system HapDefX capable of detecting multiple normal forces on a large 3D robot's limb surface using a sparse sensor configuration, as shown in Figure 1. We tackle the problem of reducing energy consumption and increasing data acquisition rate by opting for a small number of optimally placed physical sensors, which measure the internal deformation. In addition to the high resolution for single-contact points, this system can robustly localize up to four contact points and predict the respective force magnitude.

The contributions of the paper are the following:

- proposal of a new way of implementing a 3D multi-contact haptic sensor;
- a machine learning framework for inferring contact location and normal forces for multiple-contact points using insufficient real multi-contact data;
- implementation of the proposed system on a robotic limb.

The paper is structured as follows: section 2 reviews the related work. Section 3 presents the method by first giving an overview and then investigating the different aspects from single- to multi-contact prediction. In section 4, we present the results on the robotic limb. We close with a discussion in section 5.

## 2. Related Work

In order to make it easier to understand state-of-the-art large surface haptic applications, we gathered a representative set of approaches: array shaped sensors, optic sensors, anisotropic electrical impedance tomography (aEIT) based sensors and sensor systems with sparse sensor configuration.

HEX-o-SKIN by Mittendorfer and Cheng (2011) integrates a proximity sensor, an accelerometer, three normal force sensors and a temperature sensor on one 15 × 15 mm hexagonal printed circuit. It allows covering a surface, e.g., of a robot exoskeleton, with multiple HEX-o-SKIN chips forming a dense array. In this way a large surface can be covered, however, the robustness of the system might be challenging.

TacCylinder by Ward-Cherrier et al. (2018) is a camera-based system. It is shaped as a cylinder with a tube through its center, which holds a camera and a bulky catadioptric mirror system to capture the whole limb deformation pattern internally. The sensor has a dimension of 63 × 63 × 82 mm and delivers comprehensive information about the deformation of the soft cylindrical surface. The surface shape is restricted and a new shape requires an adaptation of the optical system. Additionally, the inside of the robotic part needs to be empty for this method to be applicable.

Lee (2017) uses stretchable conductive materials (skin) with a few electrodes assembled on the skin boundary and measure all combinations of pairwise conductivities. The force location is determined by anisotropic electrical impedance tomography (aEIT). Only 16 electrodes are required on the skin boundary with skin size of 40 × 100 mm. However large computational costs arise, requiring special hardware.

In a previous work, we proposed HapDef (Sun and Martius, 2018), which employs Machine Learning for single-contact force prediction from a sparse sensor configuration. With this method contact position and force magnitude can be inferred with sufficient precision on a robot shin with a surface of about 200 × 120 mm equipped with only 10 strain gauge sensors (8 × 5 mm each). The positions of the sensors are optimized using different optimization criteria. Using the same physical setup and taking it as a basis, we explore in this paper the potential of the setting for more precise measurement and the extension to multiple-contact points. We will elaborate in section 3 on more details about the HapDef design choices.

To put the multi-contact tactile spatial accuracy in relation, we compare it with the acuity of human tactile sensation quantified by the “two point discrimination” criterion, which is widely used to assess tactile perception in clinical settings (Shooter, 2005; Blumenfeld, 2010). It describes the ability to distinguish two nearby stimulations on the skin to be two distinct contacts instead of one. In the human body, this ability largely differs from body part to body part (Bickley et al., 2017). We will compare to the acuity on the fingertip, palm and shin.

## 3. Methods

We propose a method to obtain a robust, multi-contact, large-surface haptic device from a few sparsely placed deformation sensors. The key ingredient is that we use Machine Learning methods to infer the interaction forces from the sensor readings. In Sun and Martius (2018), such approach was already used to find an optimal placement of the sensors and to infer single-contact information. In order to tackle the multi-contact scenario, we propose to use a new pipeline using neural networks.

For a better understanding of the problem, we elaborate on the hardware setup and some of the design choices. In order to obtain a system with whole surface haptic sensation and with high robustness to interactions, we decided to pursue an approach where sensors are placed inside a deformable shell. The shell is around a structural part, in our case of a robot limb, which has an inner support structure. In our case the inside is mostly hollow to be lightweight and allow for a easy assembly of strain gauge sensors. The surface also contains holes for several reasons: firstly, to simplify the assembly, and secondly, to improve the localization by breaking the symmetry of the deformation pattern and limiting the deformation effects. A more systematic study of the best surface shape remains for future work. In principle, our method is generic and can be applied to other hardware configurations with arbitrary shapes. A decisive property of the hardware needs to be that force interactions yield a stimulation of multiple sensors, which can be typically achieved by adjusting the softness of the shell. A result of the sparse sensor configuration is that sensor values do not directly correspond to a force and location of a haptic stimulus, which instead need to be inferred.

We start with the problem description. Given sensor readings from a small number of real physical sensors $R\in {\mathbb{R}}^{N_{R}}$ (here *N*_*R*_ = 10), we want to infer one of two quantities, where both can be used to describe the multi-contact information. The first one is the deformation map of the entire surface represented by the displacement at many points $D\in {\mathbb{R}}^{N_{D}}$ (*N*_*D*_ = 3, 211 here) with *D*_*i*_ = 0 indicating no displacement at the particular point. This representation is similar to a visual input representing the interaction forces by a pattern. The second quantity is the explicit contact point information, i.e., the positions $P_{i}\in {\mathbb{R}}^{3}$ and their respective force impact *A*_*i*_∈ℝ for all of the unknown *T* contact points *i*∈[1, …, *T*]. This representation is much more direct and low-dimensional and allows for a quantitative comparison in terms of position and force (magnitude) accuracy but it is more difficult to obtain.

**Figure 2:**
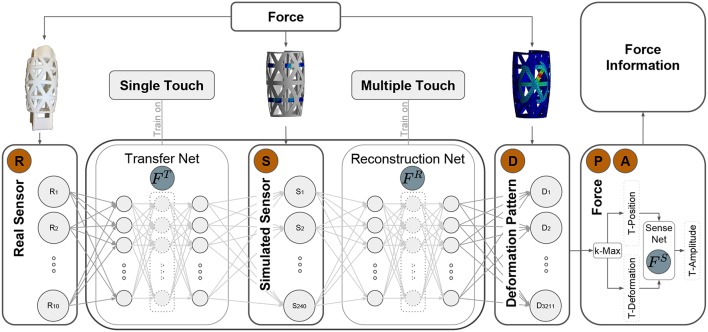
General framework for multi-contact information inference: the framework first correlates the 10 real physical strain gauge values with the simulated sensor values using Transfer Net *F*^*T*^ and then predicts the deformation pattern based on the simulated sensor values using Reconstruction Net *F*^*R*^. According to the predicted deformation pattern, the *T* multi-contact's candidate locations are extracted using k-Max method. Depending on the rigidness distribution of the 3D structure, the force magnitude of each candidate contact is predicted based on the location and the deformation using Sensitivity Net *F*^*S*^. All predicted candidate force information is then summarized into robot system for further post-processing to cancel out the spurious contacts.

In order to implement the inference machine for the above task, a large amount of data is required. In Sun and Martius (2018), a physical probing device is proposed which allows to automatically collect data for single-contact points. Performing this with multiple-contact points is very challenging. One would need to stimulate the system in a controlled manner with multiple independent force tips. More than one robotic arm would be required in order to automate this process. Apart from the hardware effort, their control would need to avoid collision and so forth. We propose, instead, to exploit finite element simulations for simulating the deformation patterns for multiple contact points. This allows us to collect large amount of data linking contact points to displacements of the surface. How to link this to the real system? For the real system, we use the data for single-contact points. The hypothesis is that, after learning the relation between real sensor values and simulated sensor values, we can then predict the deformation patterns for multiple contact points. It remains to predict the position and force magnitude for each contact point from the deformation pattern. Our architecture is presented in Figure 2.

The method decomposes into three sub-tasks: transfer learning from real to simulated sensors (Transfer Net *F*^*T*^), reconstruction of the deformation pattern from simulated sensors (Reconstruction Net *F*^*R*^), and the position and force magnitude prediction (position and number of contacts detector *k-Max* and Sensitivity Net *F*^*S*^). Each of the neural networks can be trained using its own training scheme and dataset which we explain in the following.

### 3.1. Transfer Learning

The transfer learning is implemented by the Transfer Net *F*^*T*^ mapping real sensor values *R* to simulated sensor values *S*. In order to obtain the simulated sensor values, the model of the structure (here the robotic limb) is simulated with a finite element simulation tool, ANSYS by Lawrence (2011). For a virtually applied force, the displacement of the surface can be calculated. The real sensors are strain gauge sensors that measure elongation. Since they are applied on the inside of the skeleton shell, an elongation occurs if the surface is bent. However, in simulation we cannot measure such bending directly, as we do not have the corresponding sensors but we can recover the displacement information solely from the individual points. We can solve this problem by defining a “virtual sensor" as a patch of points around the physical sensors, as shown in Figure 5B. In our case, for each physical sensor we have a patch of 24 points, as explained in section 4.2. The training data is obtained using a probing device as proposed in Sun and Martius (2018). This device is a modified 3D-printer where the print head is replaced by a force-tip measuring the interaction force. There is an additional motor to rotate the structure, such that all points on the surface can be probed. From this setup, we obtain data containing real sensor readings and corresponding force position and magnitude. The target values for the transfer net, i.e., the simulated virtual sensor values, are obtained by applying the same forces in the simulation and minimizing the mean squared error:

(1) $$L^{T}(\theta )=E\left[{\left\|F_{\theta }^{T}(R)-S\right\|}^{2}\right]\text{​}.$$

### 3.2. Reconstruction of the Deformation Map

To infer the whole deformation map, i.e., the displacements at all points on the surface, we use the Reconstruction Net *F*^*R*^ mapping *S* to *D*. The data for training this network is obtained solely from simulations for different numbers of contact points and force magnitudes.

The minimization objective is again the mean square loss:

(2) $$L^{R}(\theta )=E\left[{\left\|F_{\theta }^{R}(S)-D\right\|}^{2}\right]\text{​}.$$

### 3.3. Force Position and Magnitude Prediction

If we want to obtain the number and the position of the interaction points, we need to predict this from the deformation pattern. Intuitively, we expect the displacement to be locally maximal for the points where the forces apply. This is also consistent with studies on thin plate deformations by Ventsel et al. (2002), where the displacement at the contact force location is the highest and decreases with the distance approximately following a bell shape. Thus, the local maxima have to be detected, which we do with a simple *k*-nearest neighbors algorithm called k-Max. For each point on the surface, the displacement of the *k* nearest neighbors is compared to its own displacement. If the point has the largest displacement among the neighbors it is extracted as a candidate force location. The number of neighbors specifies a spatial region around 10 mm radius within which we only extract one maxima.

**Figure 3:**
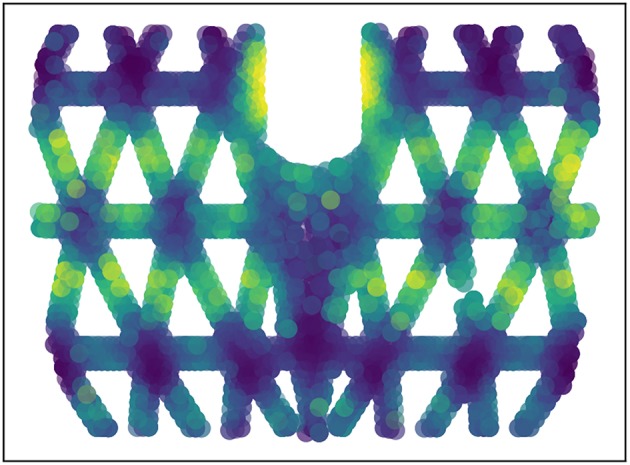
Rigidness analysis of the 3D structure: the same force is applied on each point on the surface and the displacement is compared. Lighter colors indicates bigger displacements and less rigidity (normalized for visibility).

The rigidity of the surface might vary from point to point, such that we learn the mapping from displacement to force magnitude depending on the position with the Sensitivity Net *F*^*S*^. This is trained using the squared-error loss:

(3) $$L^{S}(\theta )=E[{\left\|F_{\theta }^{S}(D_{i})-(A)\right\|}^{2}],\text{​}$$

where *D*_*i*_ refers to the displacement at the location of contact and *A* corresponds to the respective force (magnitude). The data is again collected from simulations. Note that in this case the algorithm might find more contact points than there are in reality due to non-linear surface deformations. The rigidity of the surface we study here can be visualized using *F*^*S*^ with a constant force input, see Figure 3. The upper and lower parts connected with the thick joint boundaries are the most rigid areas of the surface, as well as the hexagonal connection hub.

### 3.4. Architectural Choices

We optimized some aspects of the architecture in a systematic way.

#### 3.4.1. Real Sensors and Transfer Net

The number and the placement of the physical sensors was optimized using a greedy strategy to maximize single-contact performance as in Sun and Martius (2018). The transfer learning *F*^*T*^ is chosen to be a fully connected Feed-forward Neural Network (FNN) (6 layers with 250 tanh hidden units each, trained on 27,000 data points (*R, S*) with 60% for training and 20% for validation and testing. The number of layers and the units are selected via Bayesian hyper-parameter optimization.

In theory, for analyzing the generalization property of relationship between real physical sensor and simulated sensor, each real sensor could have been trained independently. But in order to allow for the inhomogeneous rigidness of the 3D structure, the network connects all real sensors to all simulated sensors. The number of simulated sensors is important for the generalization performance in the case of multiple contact points. On one hand, from the perspective of multi-contact prediction from simulation, the number should be chosen as high as possible. The extreme case would be the number of simulated sensor points is equal to the number of deformation points on the surface. This would make the reconstruction step unnecessary. On the other hand, we want the entire system to generalize from single-contact data during training of the transfer net *F*^*T*^ to multi-contact without retraining it, because we have no data to do so. This implies to use only a small number of simulated sensors, because the transfer learning should really just capture the mapping from real sensors to the simulated patches around each sensor individually. This enables the generalization to multiple contact points where the sensors are differently correlated. Since the physical sensor and the simulated sensor measure different quantities, we have chosen a small patch around each physical sensor location reflecting roughly the surface of the physical sensor, as displayed in Figure 5B. The number of points/the size of the patches are optimized hyper-parameters.

#### 3.4.2. Reconstruction Net

The architecture of the Reconstruction Net *F*^*R*^ is chosen to be a FNN with 600–1,200–1,800 ReLU hidden units as a result of hyper-parameter optimization using validation data of single-, double-, triple- and quadruple-contact.

#### 3.4.3. Force Position and Magnitude Prediction

For finding the contact point in the deformation map, we also considered different algorithms, such as Gaussian Mixture Model (GMM) (Bishop, 2006) and fitting Radial Basis Functions (RBF). Since the task is not a density estimation, GMM cannot be applied directly and the data would need to be transformed. For fitting the RBFs a good initialization is needed (Anifowose, 2012) in order to obtain consistently good results. The nearest neighbor approach k-Max we propose shows superior performance in our setting.

The Sensitivity Net *F*^*S*^ is a FNN with four layers, 250 hidden ReLU units mapping contact location and deformation to the force magnitude. It is trained on 27,000 data points (local deformation, position → A) with 60/20/20% training/validation/testing splitting.

## 4. Results

Before presenting the results for multi-contact sensation, we will first compare our approach using neural networks for single-contact prediction with the previous work (Sun and Martius, 2018). Then we will investigate the performance of different parts of our architecture related to the multi-contact generalization. Afterwards, the capabilities of the proposed system HapDefX is tested on up to four contact points.

### 4.1. Direct Single-Contact Prediction Baseline

**Figure 4:**
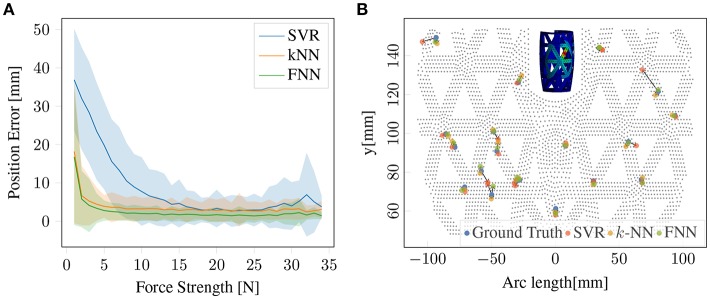
Single-contact information prediction baseline: **(A)** Prediction accuracy for contact position using SVR, *k*-NN and FNN. **(B)** Comparing predictions of SVR, *k*-NN, and FNN for single-contact force information in a 2D surface projection. Gray dots represent geometry grid, blue dots are ground truth force positions, red, orange and green dots are predicted force positions using SVR, *k*-NN, FNN, respectively. Arrows indicate the error vectors.

The predicting of the position and force magnitude of a single-contact point can be achieved with a simple regression model from real sensor values (*R*) to force position and magnitude (*P, A*). In Sun and Martius (2018) this was done with support vector regression (SVR). We will first present results on using *k*-nearest neighbors (*k*-NN) regressor and a FNN for this task. The results are presented in Figure 4A. In contrast to *k*-NN and SVR, which predict each coordinate independently, the FNN makes joined predictions allowing it to exploit correlations in the output space. As the results show, the FNN is outperforming *k*-NN with a small margin and SVR with a big margin in this task. Figure 4B illustrates the predictions for random contact positions and forces. Thus, the FNN model seems to be a suitable architecture for this regression problem. This FNN method is used as an optimal baseline for the single-contact performance.

The hyper parameters for three methods are optimally chosen based on 27,000 training data samples split as above. SVR: *C* = 20, ϵ = 10^−6^, γ = 2^−3^; *k*-NN: *k* = 6 nearest neighbors, weighted by distance; FNN: 5 layers with 500 ReLU units each.

### 4.2. Generalization of Deformation Prediction on Multi-Contact

**Figure 5:**
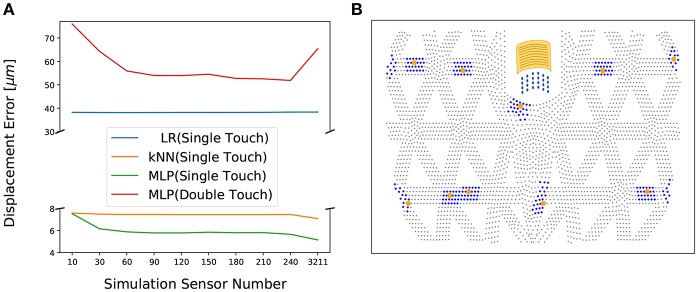
Transfer learning maps the physical sensor values to simulated sensor values: **(A)** The effect of number of simulated sensors on the prediction precision (of the full displacement map) evaluated with three methods: Linear regression (LR), *k*-NN, and FNN (*F*^*T*^). Training was conducted on a large set of single-contact data and the reported test performance is for single-contact and double-contact data collected on the real system. The reconstruction net *F*^*R*^ was trained on simulated multi-contact data. Twenty-four sensors per physical sensor yielding 240 sensors is optimal for generalizing to double contact prediction. **(B)** The yellow dots are the centers of physical SGs (optimally selected in Sun and Martius, 2018) and the yellow sheet illustrates the real physical SG. The blue dots around each SG's center are the simulated sensor points (nodes in finite element simulation ANSYS).

In order to be able to infer multi-contact force information without having sufficient training examples from the real system, we resort to transfer learning. As illustrated in Figure 2, the real sensor values should be mapped to simulated sensors, because we can train a model from simulated sensors to the full deformation map using simulated multi-contact data. The design choice is now how many simulated sensor around each physical sensor should be used? In Figure 5A, the performance of single- and double-contact prediction depending on the number of simulated sensors is evaluated. The transfer learning part is trained using single-contact data only (27,000 points). Test performance is based on single- and double-contact data from the real system. The optimal number is suggested to be 24 simulated sensor per strain gauge (SG) resulting in 240 sensors, because this yields the best performance on double-contact. Their arrangement is around the center of the physical SG as shown in Figure 5B.

After the simulated sensor arrangement is decided, we can evaluate the capabilities of the reconstruction network *F*^*R*^ for inferring the right displacement map. For this we generate data in simulation for different number of force-points impacting the surface. We have single-, double-, triple- and quadruple-contact with randomly selected contact positions and forces (each type with 27k, 6k, 6k, 6k samples with the usual training, validation, testing splitting).

We evaluate the performance of different models for the reconstruction process, namely linear regression (LR), *k*-NN, and FNN (*F*^*R*^). Table 1 shows the results for the accuracy of predicting the global deformation map depending on which data is used for training and testing. Two essential messages can be extracted: First, linear regression is best at generalization from only single-contact data to multi-contact prediction, however results are poor. Secondly, the FNN performs best when data for the respective number of contact points was present during training and it also generalizes best from single-, double-, and triple-contact data to predicting quadruple-contact. Thus, using training data for multiple-contact points is clearly beneficial. In the following we will use the FNN for *F*^*R*^ trained in all contact data.

**Table 1:** Reconstruction task: comparison of multi-contact prediction methods and dataset for predicting the displacements *D* from simulated sensors *S*.

**Displacement Prediction Error [**μm**]**																	
LR	Train	I				I and II				I, II, and III				I, II, III, and IV			
		2.2				6.2				10.9				13.6			
	Test	I	II	III	IV	I	II	III	IV	I	II	III	IV	I	II	III	IV
		2.2	36.6	60.3	78.2	1.9	16.1	24.4	31.8	2.0	15.6	23.0	29.8	2.1	15.6	22.6	29.0
*k*-NN	Train	I				I and II				I, II, and III				I, II, III, and IV			
		2.3				12.8				27.5				40.9			
	Test	I	II	III	IV	I	II	III	IV	I	II	III	IV	I	II	III	IV
		2.3	159.0	225.1	286.9	2.4	36.3	97.8	144.6	2.3	36.8	75.8	109.6	2.3	37.8	73.36	99.8
FNN	Train	I				I and II				I, II, and III				I, II, III, and IV			
		0.6				2.9				5.7				8.1			
	Test	I	II	III	IV	I	II	III	IV	I	II	III	IV	I	II	III	IV
		0.6	82.3	123.6	158.1	0.7	7.7	30.6	48.6	0.8	7.0	15.5	25.7	1.1	6.8	13.4	20.2

*I, II, III, IV stand for dataset of single-, double-, triple-, and quadruple-contact, respectively. Gray columns indicate extrapolation to unseen number of contact points*.

**Figure 6:**
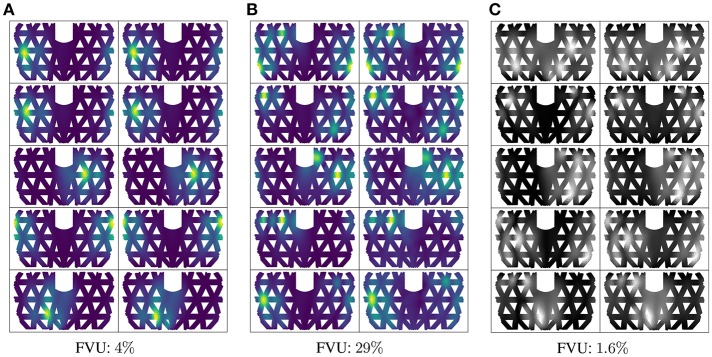
Deformation prediction results: **(A,B)** are deformation patterns of single- and double-contact based on the data collected from the real system. Left columns show ground truth (simulation: S to D) and right columns the prediction for random samples (real: R to S to D). **(C)** shows predicted deformation patterns of triple contact based on simulated sensor values (S to D).

A visual comparison between simulated and predicted deformation maps are displayed in Figure 6. They show a remarkable coherence. For single- and double-contact, the prediction is based on real physical sensor values. Limited by hardware, real sensor values for triple- and quadruple-contact can not be validated, Figure 6C shows the results for the reconstruction net *F*^*T*^ using simulated sensor values. It shows that the method is very robust. In terms of prediction error on the displacements, we evaluate the method using the normalized fraction of variance unexplained (FVU). A value of zero corresponds to perfect prediction and a value of 100% indicates the prediction is as bad as predicting the mean of targets. The FVU shows the prediction of the displacement is quite accurate, achieving 4% for single-contact and 29% for double-contact. However, these numbers do not explicitly tell how accurately the position and force magnitude of the individual contact points can be detected. This we will investigate next.

### 4.3. Force Location and Magnitude Prediction

**Figure 7:**
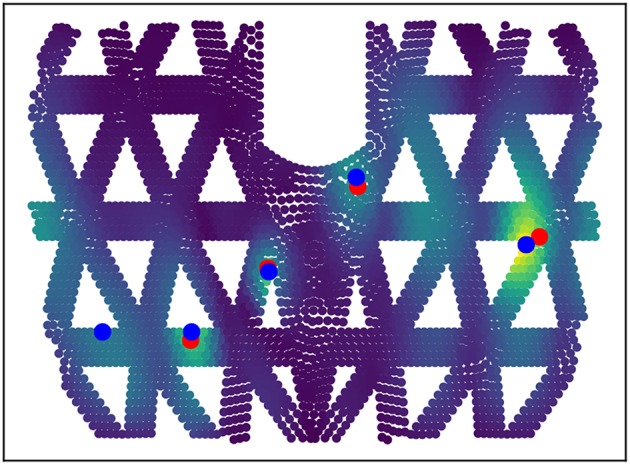
Quadruple contact information extraction. Based on the predicted deformation map (lighter color indicates stronger deformation), the multiple contact locations are extracted using the proposed k-Max method. Blue dots are the predicted contact locations and red dots show the ground truth.

Based on the predicted deformation patterns, the locations of the contact points can be extracted using k-Max, see section 3.3. In Figure 7, the force locations for four contact points is extracted, where the predicted locations show a small offset w.r.t. the position of the actually applied forces. This is due to the fact that, for specific geometry, the biggest displacement tends to be on the beam bounds.

**Figure 8:**
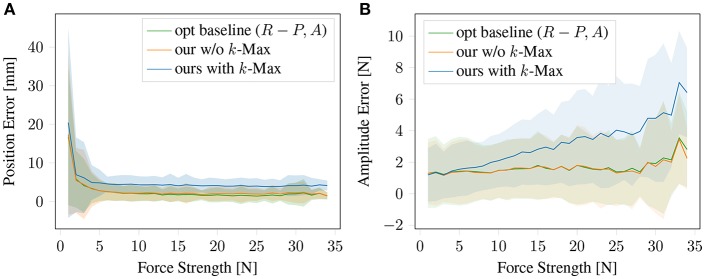
Force position and magnitude prediction on single-contact data. Comparing our architecture with the method only usable in the single-contact case: the optimal baseline mapping directly from sensors to the target and our entire framework without k-Max but instead predicting *P, A* from *D* directly. **(A)** Contact position prediction error and **(B)** Force magnitude prediction error.

The prediction precision of single-contact force location using k-Max gets slightly worse than direct prediction using FNN directly as shown in Figure 8A. In this graphics we ablate different parts of the architecture by bypassing the simulated sensors or other parts. The results show that for single-contact the additional parts that are needed for multi-contact do not harm the performance. Note that the direct prediction of the force position and magnitude is not possible for multiple contact points. As consequence of the 3D curved limb structure, we can get candidate predicted force location, which is shown as blue dot without a corresponding force application. However, when computing the predicted force magnitude for all candidate points, the spurious ones have typically by far the lowest magnitudes.

In order to predict the magnitude of the force, the rigidity of the surface has to be taken into account, see Figure 3. The same force will cause different displacements at different points. This is captured by the Sensitivity Net *F*^*S*^, which takes the position and the deformation at the detected peak displacement into account and predicts the magnitudes for each potential force position. As shown in Figure 8B, the prediction precision of single-contact force magnitude using k-Max is getting worse with increased force strength, the relative error is still low. This performance drop can potentially be avoided if the sensitivity net is trained jointly, to compensate for systematic errors in the force-location detection, for instance. The performance of the k-Max procedure is slightly lower than the direct methods, but it can be used for predicting multiple contact points.

### 4.4. Testing the Whole Framework

With the full pipeline in place, we can evaluate the method on single- and double-contact data collected from the real system and quantify its performance in an interpretable way. In order to test the multi-contact performance, we collect a double-contact dataset on the real system for 270 pairs (with a distance of 12–75 mm) of contact points with 25 different amplitudes each. One contact point has a fixed force around 30 N (by the robot arm) while the other contact point varies from 0 to 34 N (manually applied with a force tip).

**Figure 9:**
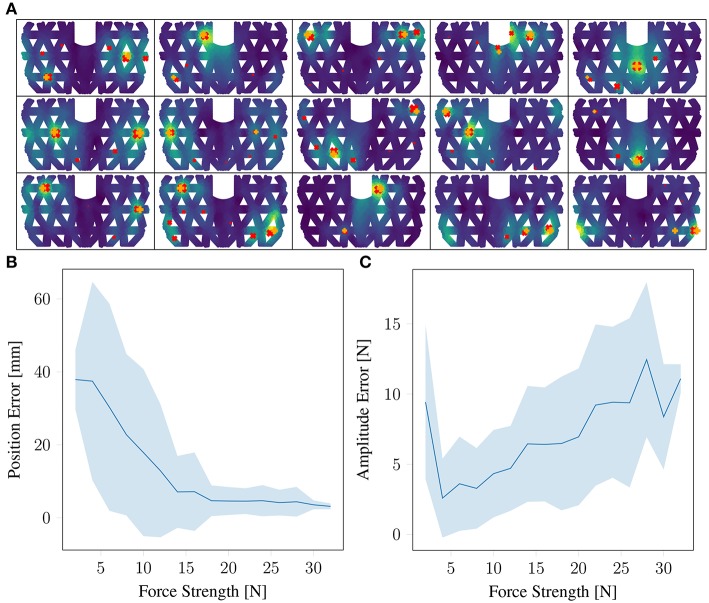
Results on double-contact of the entire framework: **(A)** are predicted deformation patterns and detected contact points (orange +: true points, red ×: detected) for double-contact. The size of the crosses reflect the force strength. **(B)** is the prediction error for position of double contact based depending on the force strength. **(C)** is the same for force magnitude. One contact points is fixed at around 30 N.

The above mentioned force extraction method yields a set of potential contact points with their corresponding force magnitudes. As shown in Figure 9, the spurious contact points have typically significantly lower force magnitudes than the real ones, visible by marker size in the figure. For computing the distance metric for position and force prediction, we sort the found contact points by their force magnitude values and compute the average distance of the top *T* contact points with the closest real ones. *T* stands for the number of actual contact points. On average, the precision for double-contact is 14 mm for the location and 6 N for the force magnitude if we take the sorted highest *T* contacts' magnitudes as targets.

We optimize the sorting method further, as we found that often multiple contact points with similar strength are detected and spurious points often lie in between. To incorporate that, we consider the detected point *T*+1, if it exists and if it within 10% amplitude difference compared to contact point *T*. We select *T*+1 instead of *T* if this maximized the spread of the detected points. With this procedure, the precision for double-contact is optimized to 10 mm for the location and 6 N for the force magnitude as listed in Table 2. Figures 9B,C shows the prediction error in space and force magnitude depending on the force of the contact points.

**Table 2:** Prediction accuracy summary: comparison of the prediction performance of HapDefX, HapDef and Human skin.

**Precision**	**Single-contact**			**Double-contact**			
	**HapDef**	**FNN**	**HapDefX**	**HapDefX**	**Human fingertip**	**Human palm**	**Human shin**
Location [mm]	8	**3**	**5**	**10**	2–8	8–12	30–40
Magnitude [N]	1.6	**1.5**	**3**	**6**	–	–	–

*The bold numbers show accuracy improvements by the methods of this paper. Number for human skin are the “two point discrimination” distances taken from Bickley et al. (2017)*.

Our proposed HapDefX infers force magnitude information properly for robotic multi-contact applications and has a greater localization acuity than the human shin and is on par with the human palm. The comparison to the human skin is only to give an intuition and is not rigorously conducted. On the one hand, our system has holes where it is “blind” to touch, but these are not considered in the statistics. On the other hand, we compare absolute localization performance of our system with the bare ability to distinguish two stimuli measured at human subjects.

## 5. Discussion

We present a robust, low cost, large-surface haptic system with sparse sensor configuration, which is capable of sensing multi-contact location and force strength. The system is powered by a machine learning approach, which can reliably localize multiple-contact points all around a curved surface and predict the respective force strength. The example shown here uses only 10 deformation sensors covering a sensing surface of 200 × 120 mm achieving a localization precision of 5 mm on single-contact and 10 mm on double-contact. The updating rate of the hardware can be up to 1 kHz using Arduino DUE. The speed of the neural network depends on the implementation and platform, but it can also be made fast. All together, our system is only composed of low-cost components. Our method can be applied to any large-surface system where a set of sensors can be applied to a deformable surface. In fact, the deformation can and should be non-local in order to make use of the spreading of the deformation to allow for the inference process. In this paper, we also show that having training data on single-contact points is enough to detect multiple contact points for real physical system.

## Author Contributions

HS and GM conceived the method and the experiments, and wrote the manuscript. HS designed and constructed the hardware, conducted experiments, and analyzed the data. GM supervised the data analysis.

### Conflict of Interest Statement

The authors declare that the research was conducted in the absence of any commercial or financial relationships that could be construed as a potential conflict of interest.
